# Identification of nasopharyngeal microbial dysbiosis in COVID-19 patients by 16S rRNA gene sequencing

**DOI:** 10.3389/fmicb.2025.1631198

**Published:** 2025-08-29

**Authors:** Filippos S. Kardaras, Eirini Siatravani, Katerina Tsilipounidaki, Efthymia Petinaki, Artemis G. Hatzigeorgiou, Vivi Miriagou

**Affiliations:** ^1^DIANA-Lab, Department of Computer Science and Biomedical Informatics, University of Thessaly, Lamia, Greece; ^2^Hellenic Pasteur Institute, Athens, Greece; ^3^Laboratory of Bacteriology, Hellenic Pasteur Institute, Athens, Greece; ^4^Department of Medical Biopathology, Faculty of Medicine, School of Health Sciences, University of Thessaly, Larissa, Greece

**Keywords:** COVID-19, nasopharyngeal microbiome, 16S rRNA gene sequencing, nasopharynx, SARS-CoV-2, metagenomics

## Abstract

**Background:**

The coronavirus disease 2019 (COVID-19) pandemic, caused by severe acute respiratory syndrome coronavirus 2 (SARS-CoV-2), has prompted extensive research into factors influencing the onset and severity of the disease. Among these factors, the role of the nasopharyngeal microbiome, a vital ecosystem critical for respiratory health and immune modulation, remains incompletely understood. This study aimed to elucidate the relationship between the composition of nasopharyngeal microbiota and the clinical presentation of COVID-19 during the initial phase of infection.

**Materials and methods:**

A total of 81 nasopharyngeal swab samples were collected from individuals in Central Greece between January and February 2021. Following quality control, 77 samples were selected for microbiome analysis. This selection included SARS-CoV-2-negative controls (NE, *n* = 26) and SARS-CoV-2-positive patients classified as asymptomatic (AS, *n* = 19), mild (MI, *n* = 16), or severe (SE, *n* = 16) based on clinical criteria. All COVID-19-positive samples were collected within 2 days of symptom onset, and participants with recent hospitalization or antibiotic use were excluded. Microbiome profiling was performed using 16S rRNA gene-targeted metagenomic sequencing, followed by comprehensive bioinformatics and statistical analyses.

**Results:**

Significant differences were observed in both alpha and beta diversity measures, with alpha diversity decreasing as the severity of COVID-19 increased. Three of the four individual study groups, namely NE, MI, and SE, exhibited distinct microbial profiles, while the asymptomatic group showed greater heterogeneity. Significant variations in the abundance of specific phyla, families, and genera were identified between the different study groups. When comparing the NE and SE groups, we observed a significant increase in the abundance of the Proteobacteria phylum in the SE group, while the abundance of Fusobacteria was significantly lower in the SE group. In symptomatic COVID-19 patients, we observed a significant reduction in the abundance of key family constituents of the nasopharyngeal microbiota, such as Fusobacteriaceae, Prevotellaceae, and Streptococcaceae, suggesting a disruption in microbial homeostasis during the infection. Conversely, we found an increased prevalence of families associated with pathogenic or opportunistic pathogenic bacteria, including Enterobacteriaceae and Bacillaceae, in the SE group, suggesting a potential role of these taxa in the disease progression of COVID-19.

**Conclusion:**

These findings shed light on specific genera that undergo significant changes during COVID-19 infection and contribute to our understanding of the dynamic nature of the nasopharyngeal microbiome in relation to disease progression and severity.

## 1 Introduction

The outbreak of COVID-19, caused by severe acute respiratory syndrome coronavirus 2 (SARS-CoV-2), has had a profound impact on public health and societies worldwide. Extensive research has been conducted on the virus, its clinical manifestations, and the immunological response; however, much remains to be understood about its interactions with the human body. In this study, we focused on the relationship between the nasopharyngeal microbiome and COVID-19, considering the nasal epithelium as a primary entry point for SARS-CoV-2 (Jackson et al., 2022).

The nasal cavity hosts a diverse community of commensal bacteria that play a crucial role in maintaining respiratory health. These bacteria help prevent the colonization of opportunistic pathogens through mechanisms such as immune modulation and competition for resources. A healthy microbiome contributes to both innate and adaptive immune responses, offering protection against invading pathogens (Huffnagle et al., 2017). However, imbalances in this microbial community may enable opportunistic pathogens to proliferate, contributing to respiratory health issues or diseases.

The possible influence of changes in the nasopharyngeal microbiome on COVID-19 might depend on how these alterations modulate the host’s immune response to the virus, possibly amplifying or dampening the host’s reaction to SARS-CoV-2. Notably, altered microbiota may suppress the activation of host defense signaling, potentially heightening the susceptibility to SARS-CoV-2 infection and its associated pathogenesis. For instance, respiratory pathogens, such as *Klebsiella pneumoniae* have been shown to limit the activation of NF-κB-driven responses, which are intrinsic to the host’s antiviral defense mechanisms (Bengoechea and Sa Pessoa, 2019). Furthermore, interferons of types I and III, which are produced in response to bacterial infections, could potentially facilitate SARS-CoV-2 infection, as the virus uses the ACE2 receptor, an interferon-stimulated gene (Hoffmann et al., 2020); however, the actual implications of interferon-mediated ACE2 upregulation on virus entry and infection remain unclear.

Numerous studies have investigated the relationship between the nasopharyngeal microbiota and COVID-19; however, the results have been highly inconsistent, partly due to differences in study design, participant age, and other factors, as reviewed in the 2022 study by Merenstein et al. (2022).

In the present study, we analyzed the nasopharyngeal microbiota in various groups of Greek COVID-19 patients (specifically those with asymptomatic (AS), mild (MI), or severe (SE) infections) at the initial stage of infection. We compared their microbiota composition with that of SARS-CoV-2-negative individuals using 16S rRNA gene-based targeted metagenomics. The specific microbiota patterns identified among the COVID-19 patient groups were correlated with the severity of the infection.

## 2 Materials and methods

### 2.1 Patient groups and sampling

A total of 81 nasopharyngeal swab samples obtained at the Emergency Unit of the University Hospital of Larissa, Greece. These samples were processed, originating from 81 individuals who had been admitted to 11 hospitals, clinics, or long-term care facilities located in Central Greece, during January–February 2021 (for patient metadata, see Additional file 1). All participants were informed about the study’s purpose and provided written informed consent. The samples were tested for the presence of SARS-CoV-2 using the commercial kit “Direct SARS-CoV-2 Real-Time PCR (Vircell, Granada, Spain).”

A total of 51 participants tested positive for SARS-CoV-2 and were divided into three groups based on the maximum score reached during the day of sampling, using the 11-point WHO COVID-19 progression scale (Marshall et al., 2020): asymptomatic (AS) (*n* = 19), mild (MI) (*n* = 16), and severe (SE) (*n* = 16). Asymptomatic patients were defined as individuals with no symptoms but were tested either due to contact with confirmed positive COVID-19 cases or as a precautionary measure when entering the hospital for unrelated tests or procedures (WHO score: 1). Mild cases included patients presenting at the hospital’s emergency department with cold-like symptoms (fever and cough) but not requiring hospitalization (WHO score: 2–3). Severe cases were those who, following a positive test, were admitted to a COVID-19 intensive care unit (COVID-19 ICU) because of the severity of the exhibited symptoms such as low oxygen level and dyspnea (WHO score: 4–6). Patients in the MI and SE groups did not exhibit symptoms for more than 2 days. A total of participants tested negative (WHO score: 0) and were either hospital personnel without medical issues or hospital visitors admitted for routine preoperative COVID-19 testing. This latter group is the fourth group comprising SARS-CoV-2 negative participants (NE) (*n* = 26). Four participants (3 SE and 1 NE) were not included in the metagenomics analysis due to the low count of reads produced in the 16S experiment, as detailed in Section 2.4. In total, 77 nasopharyngeal swab samples were included in the final analysis.

Only COVID-19 patients who had not been hospitalized in the past 3 months and had not received antibacterial, antiviral, or antifungal treatment in the past month were considered eligible for the study. Participants experiencing COVID-19 symptoms for more than 2 days were excluded to ensure that all symptomatic patients were in the early phase of infection. To determine the time elapsed since infection and thereby assess the disease phase in the asymptomatic group, the anti-SARS-CoV-2 immunological status was evaluated by detecting specific antibodies against the N and S antigens of SARS-CoV-2 using commercial assays “Elecsys® Anti-SARS-CoV-2 N” and “Elecsys® Anti-SARS-CoV-2 S,” respectively, in a cobas e 602 module (Roche, Basel, Switzerland). Blood samples were obtained from AS patients at two time points, day 0 and 3 days later. AS patients whose anti-N IgG levels increased after 3 days were classified as being in the early phase of the disease, while a decrease in anti-N levels combined with an increase in anti-S antibodies indicated the recovery phase of the infection (Florou et al., 2021).

### 2.2 Nucleic acid extraction

Nasopharyngeal swabs were obtained and placed in the Transport and Preservation medium (Biocomma, Shenzhen, China). Total nucleic acid extraction was performed automatically using the NucliSENS easyMag system (bioMérieux, Marcy-l’Étoile, France) with software version 2.1.0.1. An input volume of 500 μl was used, and nucleic acids were eluted in 50 μl. The procedure followed the manufacturer’s protocol, and the extracted nucleic acids were stored at −80°C until the 16S rRNA gene metagenomic next-generation sequencing.

### 2.3 Sequencing

The extracted DNA was quantified using the Qubit fluorometer with the Qubit dsDNA HS Assay Kit (Thermo Fisher Scientific, USA). DNA libraries were prepared using the Ion 16S rRNA Gene Metagenomics kit (Thermo Fisher Scientific, USA). Briefly, extracted DNA was diluted to 1.25 ng/μl, and 12.5 ng were used in total for library preparation. Amplification was performed using two sets of primers, “V2-4-8” and “V3-6,7-9,” targeting the respective 16S rRNA gene regions that amplify the hypervariable regions of the bacterial 16S rRNA gene locus. The two sets of reactions for each sample were combined, and the DNA library was prepared using the Ion Plus Fragment Library kit and Ion Xpress Barcodes Adapters, following the manufacturer’s protocol. The libraries were quantified through qPCR using the TaqMan Universal Library quantification kit. Each library was diluted to a concentration of 100 pM and amplified. Template preparation was performed using the Ion One Touch System. Sequencing of the library was performed on the Ion Torrent S5 platform in a single-end layout.

### 2.4 Bioinformatics analysis

The Ion Torrent sequencing reads underwent quality filtering using Cutadapt v3.2 (Martin, 2011), with a quality threshold of 25, a maximum length of 300, and a minimum length of 100. The resulting quality-filtered reads were then processed using the QIIME2 pipeline (Bolyen et al., 2019). DADA2 (Callahan et al., 2016) was utilized for denoising, with the pyrosequencing option enabled (15 bases trimmed from the beginning of each read). This resulted in the identification of unique sequence variants commonly referred to as “features” (i.e., exact amplicon sequence variants representing distinct microbial taxa). A feature table, which quantifies the count of each feature in every sample, was generated (Additional file 2). Taxonomic classification of the features was conducted using closed reference OTU picking with the VSEARCH plugin (Rognes et al., 2016) and the Greengenes database (v13.8.99) as a reference (McDonald et al., 2012). Features that were not assigned to any phylum and features with a total count of one (singletons) were removed. For calculating the alpha and beta diversity metrics, a rarefaction size of 21,432 features was selected (corresponding to the fifth smallest sample), which led to the exclusion of four samples (three severe and one negative).

Statistical analysis of alpha and beta diversity metrics was performed using the QIIME2 PERMANOVA Kruskal-Wallis test (Anderson, 2001). The ALDEX2 method was applied to the 77 unrarefied samples to identify differentially abundant taxa, using we.eBH ≤ 0.05 as the significance threshold, where we.eBH represents the expected Benjamini-Hochberg corrected *p*-value of Welch’s t-test (Fernandes et al., 2013, 2014; Gloor et al., 2016). For validation, supplementary differential abundance analyses were also performed using ANCOM with default QIIME 2 settings (Lin and Peddada, 2020). Changes identified as significant by both ALDEx2 and ANCOM are highlighted in bold in Supplementary Table S1, while the complete ANCOM results are available in the associated GitHub repository. A random forest classifier was trained using the Qiime2 “sample-classifier classify-samples” method and the default parameters (Pedregosa et al., 2011; Bokulich et al., 2018). Relative abundance for each feature in a given sample was calculated as: (feature count) / (total feature counts in the sample) × 100%, representing the percentage proportion of that microbial taxon within the sample.

A visual schema summarizing the workflow from sample collection to statistical analysis is provided in Supplementary Figure S1.

## 3 Results

### 3.1 Correlation between COVID-19 severity and reduced bacterial diversity along with altered bacterial composition

**Figure 1:**
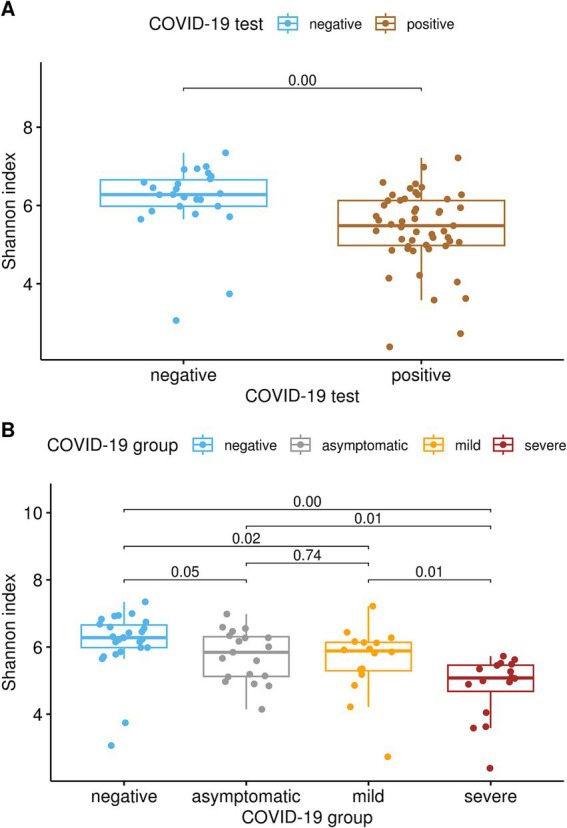
**(A)** Alpha-diversity was significantly lower in subjects who tested positive for SARS-CoV-2 than in subjects who tested negative, as indicated by Shannon’s diversity index (pairwise Kruskal-Wallis test, q < 0.01). **(B)** Alpha diversity correlated with COVID-19 severity, as severe patients exhibited significantly lower diversity compared to both the mild and asymptomatic groups (q = 0.01). Additionally, both the mild and asymptomatic groups demonstrated lower alpha diversity than the negative group (q ≤ 0.05), whereas no significant difference was observed between the mild and asymptomatic groups.

Differences were observed in both the alpha and beta diversity measures. Shannon’s diversity index (Figure 1A) was significantly decreased (Kruskal-Wallis test, *q* < 0.01) in COVID-19-positive patients compared to COVID-19-negative patients. Similarly, the alpha diversity decreased as COVID-19 severity increased. Shannon’s diversity index correlated with COVID-19 severity (Figure 1B), as the SE group showed significantly lower diversity than the MI and AS groups (*q* = 0.01), both of which had lower alpha diversity than the negative group (*q* ≤ 0.05). No significant difference was found between the MI and AS groups.

**Figure 2:**
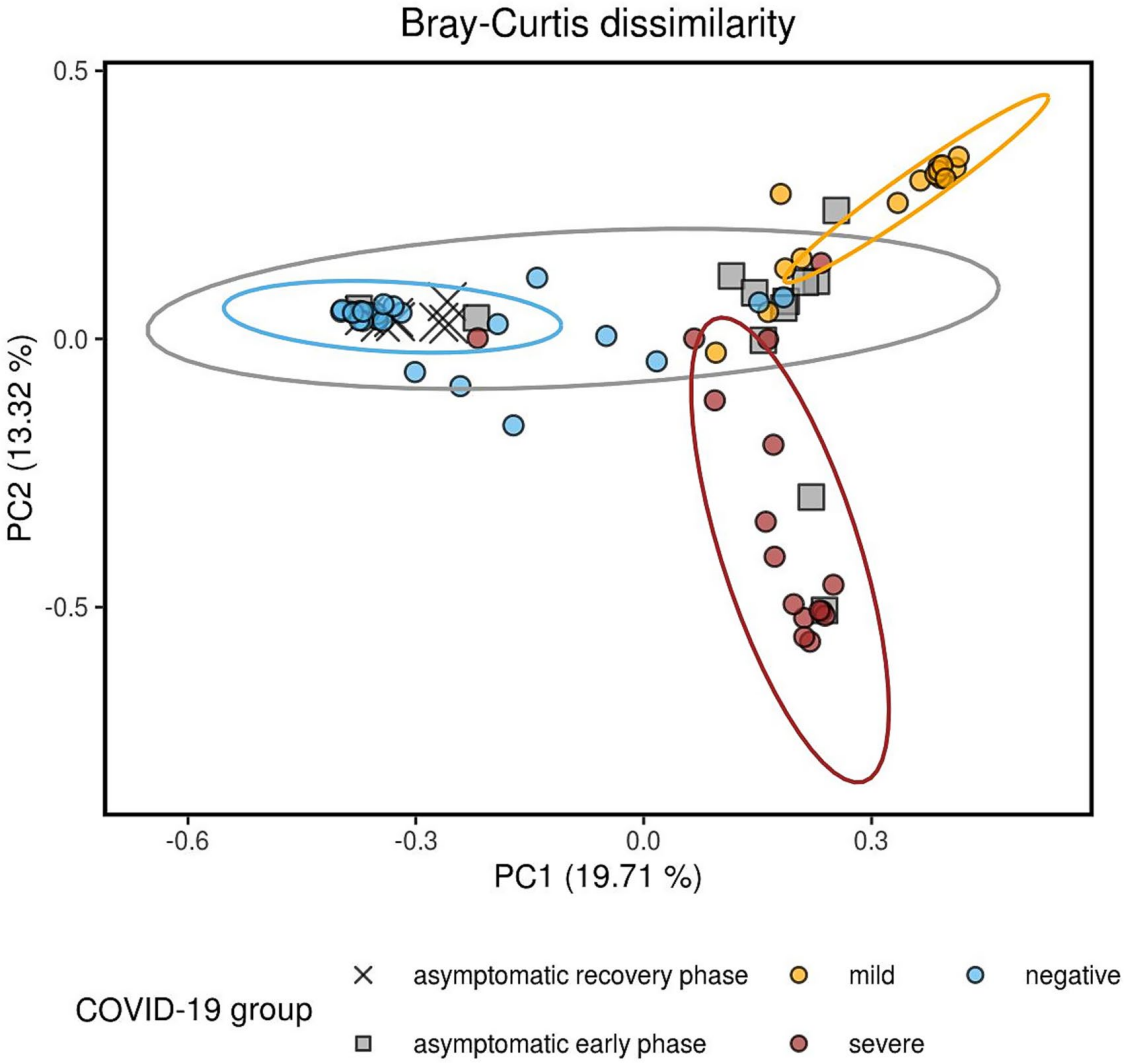
Composition dissimilarity between the four study groups, as indicated by the Bray-Curtis metric visualized using principal coordinates analysis (PCoA). Pairwise comparisons were performed using PERMANOVA, with all comparisons showing *p*-values and *q*-values less than 0.01. Data ellipses represent the 90% confidence intervals, assuming a multivariate t-distribution. The greater heterogeneity of the AS group can be explained by the anti-S and anti-N titers. AS patients whose anti-N IgG levels increased after 3 days were classified as being in the early phase of the disease, while a decrease in anti-N levels combined with an increase in anti-S antibodies was indicative of the recovery phase of the infection. All the recovery phase AS patients clustered together with the NE group.

Significant dissimilarities in composition were observed among the four groups, as shown by the Bray-Curtis (Figure 2), Jaccard, unweighted UniFrac, and weighted UniFrac beta diversity distance measures (Supplementary Figure S2) (*q*-value < 0.01 in all pairwise tests by PERMANOVA, Supplementary Table S3). Each of the four patient groups exhibited distinct microbial profiles, except for the asymptomatic group, which displayed greater heterogeneity and partial overlap with the other three groups. The observed heterogeneity in the asymptomatic patient group can be attributed to the different phases of COVID-19 disease (early phase vs. recovery phase), as determined by the examination of antibody titers against the spike (S) and nucleocapsid (N) proteins of SARS-CoV-2 over a 3-day period (Supplementary Figure 1).

### 3.2 The bacterial composition specifically characterizes each patient group

**Figure 3:**
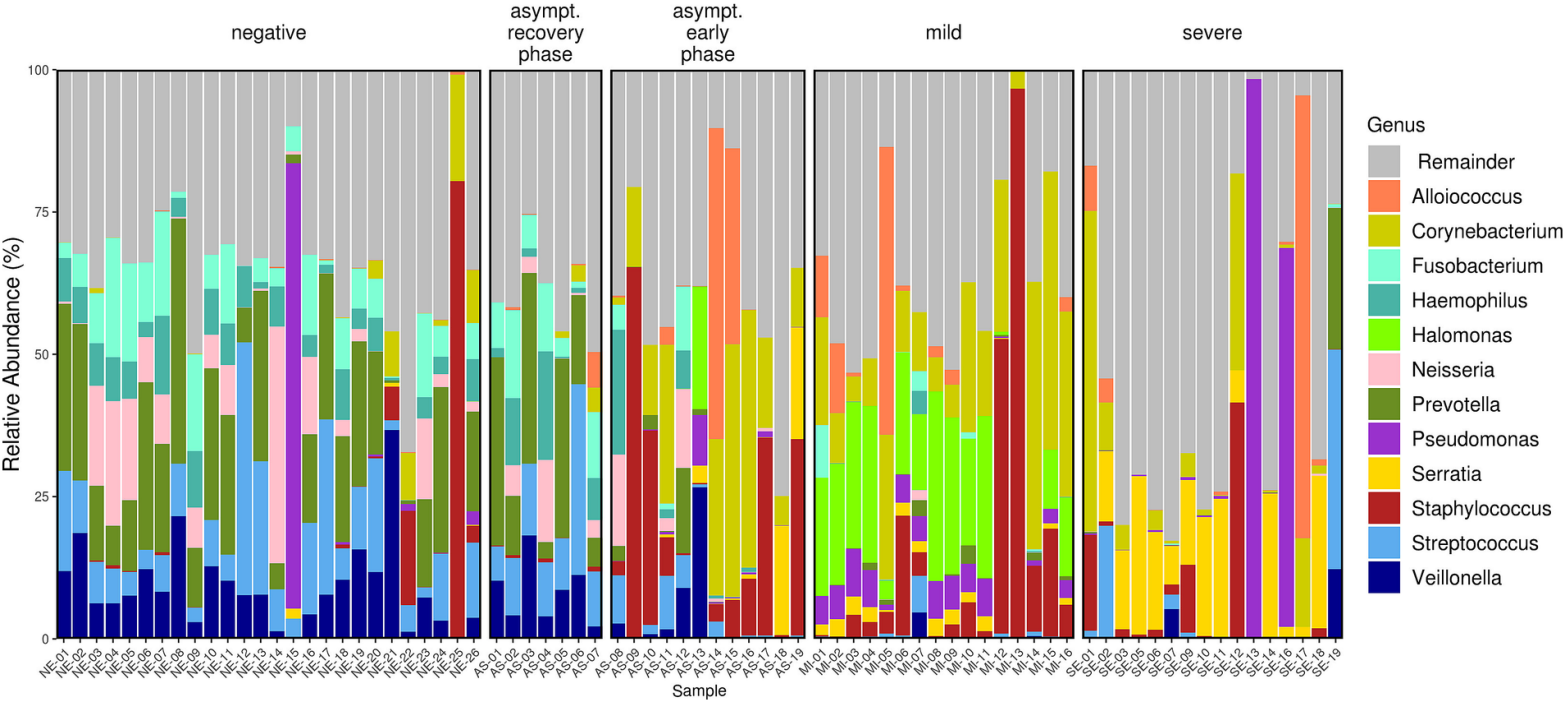
Relative abundance bar plot of the 12 most abundant genera (based on mean relative abundance across all samples). The differences in the microbiota profiles among the four groups were evident. The AS group exhibited variability, with two distinct patterns observed corresponding to the early and recovery phases of the disease.

The microbiome composition differed across the AS, MI, SE, and NE groups. The microbiome profiles at the genus (Figure 3), family, and even phylum levels were distinct for each group. The composition of bacterial genera shifted progressively from predominantly commensal bacteria in SARS-CoV-2-negative individuals to more pathogenic bacteria across the COVID-19-positive patient groups. For instance, in the NE group, the genera *Fusobacterium, Haemophilus, Prevotella*, *Streptococcus*, and *Veillonella* were predominant, and these genera are part of the nasopharyngeal microbiota. In contrast, these bacteria were found to be lower in the MI and SE groups, where they were replaced by other pathogenic bacteria, such as *Corynebacterium, Serratia,* and *Staphylococcus*.

In the AS group, two different clusters of patients can be identified. These two AS clusters align with the grouping of asymptomatic patients into early and recovery phases, with individuals in the recovery phase exhibiting similarities to the NE group. These findings show the correlation between the severity of COVID-19 and the composition of the nasopharyngeal microbiome. In addition, in the NE group, we observed two subjects (NE-22 and NE-25) with high abundances of *Staphylococci* (16.6 and 80.4%, respectively). These individuals had prior SARS-CoV-2 infections, as evidenced by their S antibody titers, which were greater than zero (57 and 62 u/ml, respectively).

To further identify the bacteria that differed in the study groups, we performed differential abundance analysis for each pairwise comparison with ALDEx2 (significant changes are reported in Supplementary Table S1, while complete ALDEX2 results are stored as Additional Files 3–5). Only early-phase asymptomatic patients from the initial AS group were included in the comparisons to ensure comparability with MI and SE patients, who were also in the early phase of infection (experiencing symptoms for no more than 2 days).

**Figure 4:**
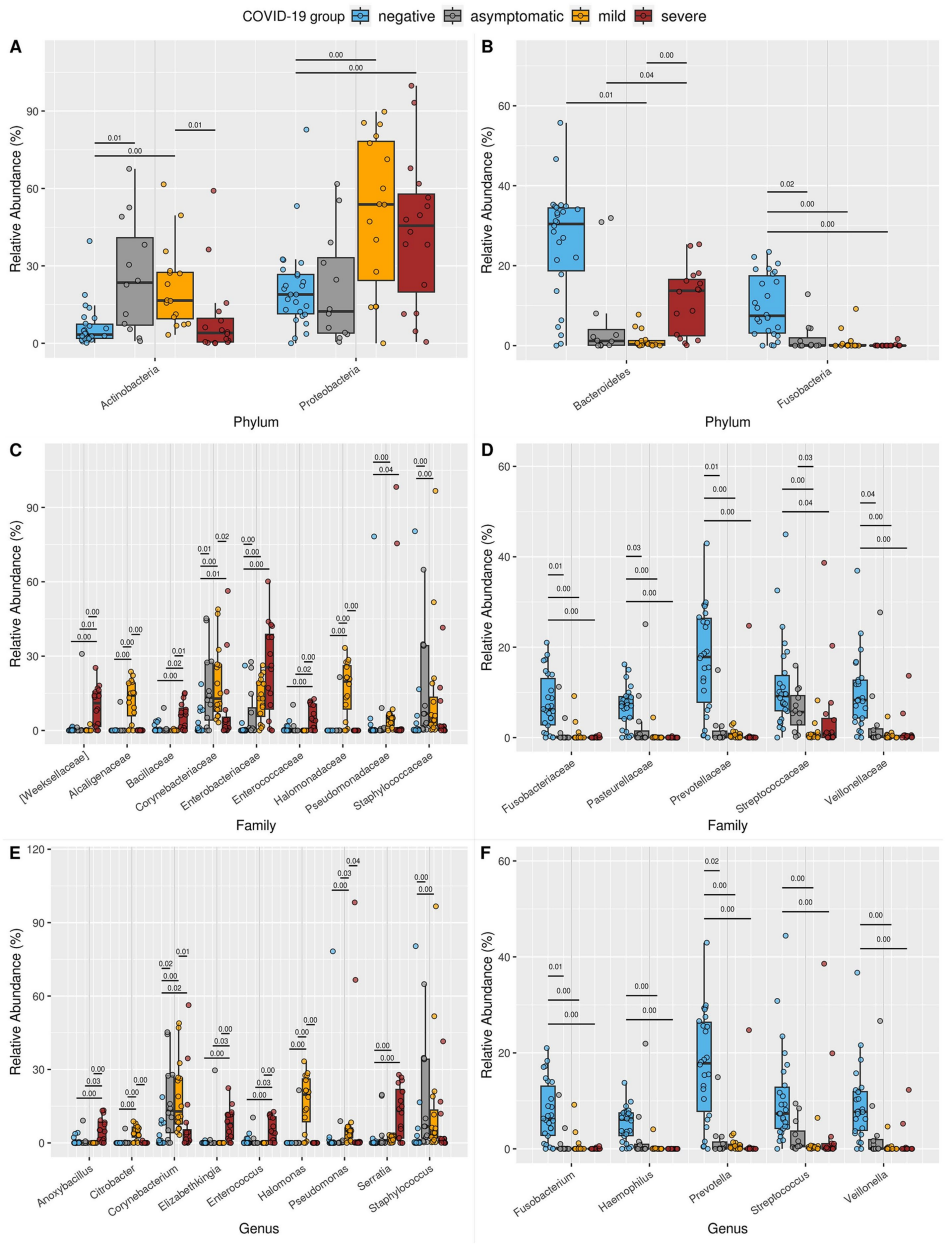
Boxplot of the relative abundance of differentially abundant taxa at the **(A,B)** phylum, **(C,D)** family, and **(E,F)** genus levels. Only taxa that were significantly different between the NE and SE or MI groups, with a median relative abundance of at least 4% in at least one study group, are shown. **(A)** Phyla enriched in SE or MI compared to NE. **(B)** Phyla reduced in SE or MI compared to NE. **(C)** Families are enriched in SE or MI compared to NE. **(D)** Families reduced in SE or MI compared to NE. **(E)** Genera enriched in SE or MI compared to NE. **(F)** Genera reduced in SE or MI compared to NE. For each taxon, all significant pairwise comparisons (Benjamini–Hochberg corrected *p*-value from Welch’s t-test, we.eBH ≤ 0.05) are annotated above the boxes: the exact *p*-value is displayed above a black line connecting the relevant groups. Supplementary Table S1 provides all exact *p*-values and groupwise median abundances. Comprehensive ALDEX2 statistical results for all taxa are available in Additional Files 3–5.

At the phylum level, when comparing the NE group with the SE and MI groups, we observed a significant overrepresentation of the Proteobacteria phylum in both the SE and MI groups (Figures 4A,B), indicating a potential role in the progression or severity of the disease. Conversely, the abundance of Fusobacteriota was notably lower in the SE and MI groups, implying a disruption in the microbial balance, which might contribute to the pathogenesis of severe COVID-19.

At the family level, a significant reduction in key constituents of the nasopharyngeal microbiota, such as Fusobacteriaceae, Pasteurellaceae, Prevotellaceae, Streptococcaceae, and Veillonellaceae, was observed in symptomatic COVID-19 patients (Figures 4C,D). This disruption of the normal microbiota may impair its protective role, creating an ecological imbalance that allows opportunistic pathogens to colonize the upper respiratory tract. Consequently, an increased prevalence of bacterial families associated with pathogenic or opportunistic pathogenic species was detected, particularly in the SE group (Weeksellaceae, Bacillaceae, Corynebacteriaceae, Enterobacteriaceae, Enterococcaceae, and Pseudomonadaceae) and the MI group (Corynebacteriaceae, Enterobacteriaceae, Pseudomonadaceae, Alcaligenaceae, Halomonadaceae, and Staphylococcaceae). These findings further support the previously described shift in microbial composition associated with COVID-19.

At the genus level, both symptomatic groups exhibited notable reductions in *Fusobacterium, Haemophilus, Prevotella, Streptococcus,* and *Veillonella* (Figures 4E,F). In parallel, the SE group showed an increased prevalence of genera known to include pathogenic species, such as *Anoxybacillus, Corynebacterium, Elizabethkingia, Enterococcus,* and *Serratia*. In the MI group, *Corynebacterium, Serratia, Citrobacter, Halomonas, Pseudomonas,* and *Staphylococcus* were more abundant. Notably, in the early phase AS group, *Fusobacterium* and *Prevotella* were enriched, while *Corynebacterium* and *Staphylococcus* were reduced compared to the NE group. Additionally, the high number of taxa differed between MI and SE, further emphasizing the distinct microbial profiles associated with these two clinical states.

These findings highlight specific genera that undergo significant changes during COVID-19 infection and enhance our understanding of the dynamic nature of the nasopharyngeal microbiome in relation to disease progression and severity.

**Figure 5:**
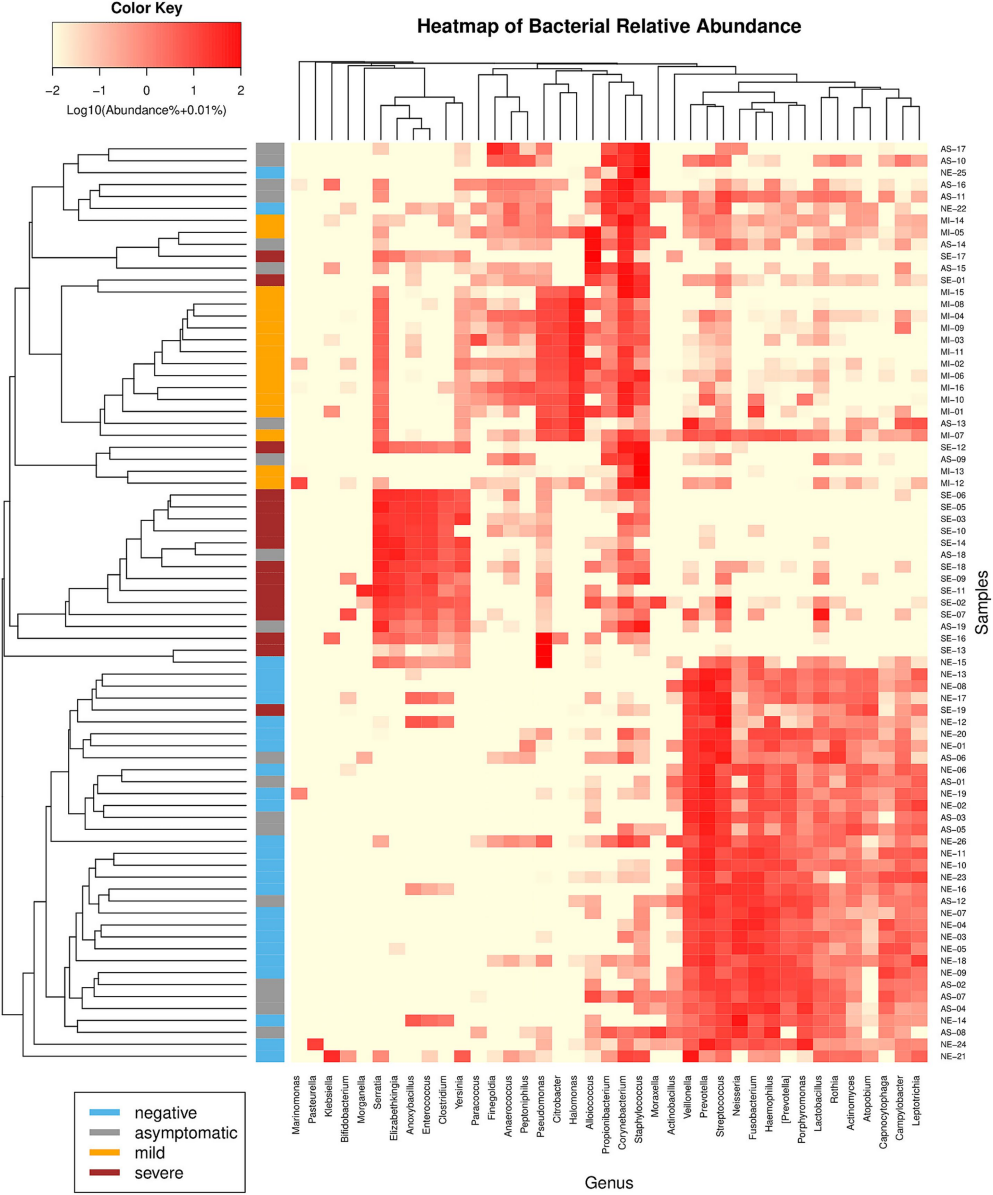
Hierarchical clustering heatmap of bacterial genera using Bray-Curtis sample dissimilarity (left) and Euclidean distance (top). Only genera constituting at least 5% of the microbiome in at least one sample are shown. The samples clustered well into their distinct groups, except for the AS patients, who presented variability.

These microbiota variations lead to the effective clustering of samples based on their respective groups when using beta diversity metrics such as the Bray–Curtis dissimilarity (Figure 5). Except for the AS group, which exhibited substantial variability, the remaining groups clustered well. This pattern underscores the distinct microbiome compositions within each group, further highlighting the unique microbial profiles associated with different states of COVID-19.

### 3.3 Random forest classifier analysis

**Figure 6:**
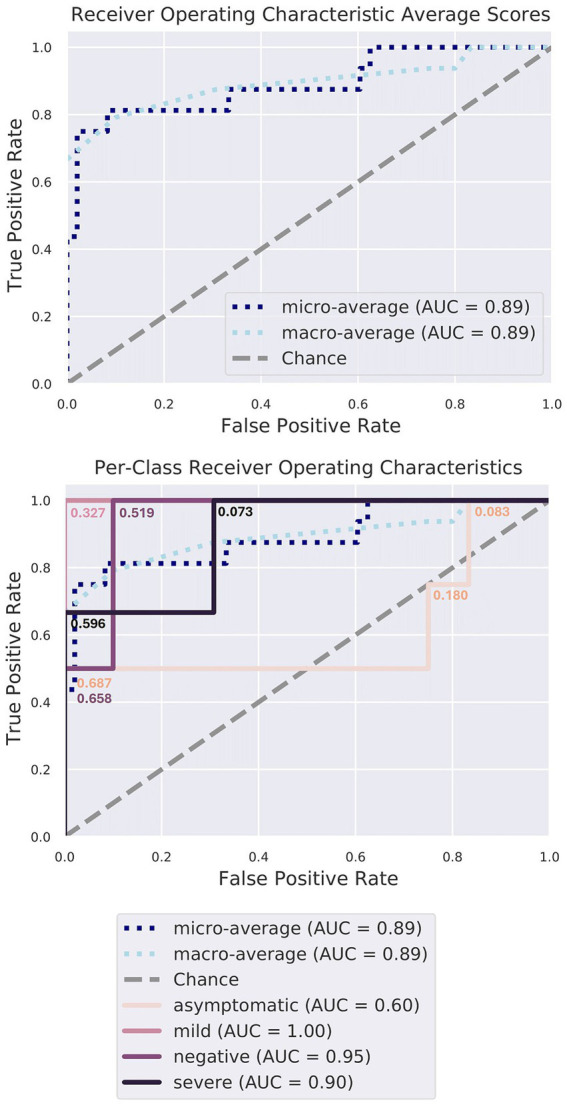
Random forest classifier model characteristics. The gray diagonal line signifies the error rate that would be expected if predictions were made purely by chance. The cutoff probability values have been annotated at each step along all four Receiver Operating Characteristic (ROC) curves, illustrating how true positive and false positive rates change as the threshold varies from 0 to 1. Notably, the curves corresponding to the MI, SE, and NE patient groups lie well above this diagonal, signifying high accuracy, surpassing random chance (area under the curve, AUC > =90%). The model excels in identifying the microbiomes of MI, SE, and NE individuals, as expected.

Random forest classifier analysis was performed to assess the significance of various taxa in distinguishing between different conditions (Figure 6). This analysis determined the discriminatory power of each genus, revealing their respective importance. The top-ranking genera in terms of importance included *Halomonas, Elizabethkingia, Renibacterium, Serratia, Oribacterium, Haemophilus, Rothia, and Corynebacterium* (Table 1; Supplementary Figures S3, S4). *Halomonas* exhibited the greatest importance, followed by *Elizabethkingia*, *Renibacterium*, and *Serratia*, suggesting their strong discriminatory power. The high relative importance of these genera underscores their role in differentiating between different disease conditions, highlighting their potential as key microbial markers for understanding and diagnosing the underlying disease states during the COVID-19 infection.

**Table 1:** Importance of the top 19 taxa in the random forest classifier model.

Genus	Importance
*Halomonas*	0.1112
*Elizabethkingia*	0.0741
*Renibacterium*	0.0687
*Serratia*	0.0649
*Oribacterium*	0.0524
*Haemophilus*	0.0496
*Rothia*	0.0406
*Mogibacteriaceae, unassigned genus*	0.0341
*Corynebacterium*	0.0235
*Streptococcaceae, unassigned genus*	0.0216
*Actinomycetales, unassigned genus*	0.0207
*Enterobacteriaceae, unassigned genus*	0.0180
*Yersinia*	0.0170
*Gulbenkiania*	0.0133
*Moraxella*	0.0130
*Alcaligenaceae, unassigned genus*	0.0129
*Rhodocyclaceae, unassigned genus*	0.0126
*Fusobacterium*	0.0114
*Pseudomonas*	0.0112

## 4 Discussion

In the present study, we characterized the composition of the nasopharyngeal microbiota in SARS-CoV-2-infected patients in Greece and compared it to that of SARS-CoV-2-negative individuals. This is the first time that this comparison has been strictly studied during the early phase of the disease, which facilitates more precise comparisons. According to our data, all SARS-CoV-2-infected patients (asymptomatic, mild, and severe) exhibited significant differences in bacterial richness (diversity indices) when compared to non-infected indviduals, which is in agreement with several previous studies (Mostafa et al., 2020; Prasad et al., 2022). Reduced diversity is often associated with a loss of beneficial commensal bacteria, which may impair the microbiome’s ability to maintain homeostasis and resist opportunistic pathogens. In several cases, the overrepresentation of *Proteobacteria* suggests a shift toward pathogenic or opportunistic taxa (Dickson et al., 2016). Interestingly, alpha diversity significantly decreased from the asymptomatic to the severe group of patients, indicating a decline in the number of different taxa and/or a less balanced distribution of taxa. Furthermore, the beta diversity analysis revealed distinct compositional shifts in the nasopharyngeal microbiota across the four groups.

The relative abundance of commensal bacteria, including *Fusobacterium*, *Haemophilus*, *Prevotella*, *Streptococcus*, and *Veillonella*, steadily declined from the SARS-CoV-2-negative group to the asymptomatic, mild, and severe COVID-19 groups. On the other hand, a notable increase in pathogenic genera was observed, with *Anoxybacillus*, *Elizabethkingia*, *Enterococcus*, and *Serratia* being prevalent in the severe group, while *Citrobacter*, *Halomonas*, *Pseudomonas*, and *Staphylococcus* were prevalent in the mild group. Several other studies have also associated SARS-CoV-2 infection with a reduction in beneficial symbiotic bacteria (Liu et al., 2021) such as *Veillonella* (Feehan et al., 2021; Gupta et al., 2022) and *Haemophilus* (Gupta et al., 2022) and an increase in opportunistic pathogens (e.g., Serratia (Feehan et al., 2021), *Moraxella* (Gupta et al., 2022), *Corynebacterium* (Gupta et al., 2022; Hurst et al., 2022), *Pseudomonas, Gemella*, and *Ralstonia* (Gupta et al., 2022). The latter pathogens could be responsible for severe coinfections of the lower respiratory tract, worsening the health status of COVID-19 patients. Our findings align with previous studies linking microbial dysbiosis to heightened inflammatory responses and poorer clinical outcomes of acute respiratory failure (Kitsios et al., 2020).

Since the patients included in the study had not been previously treated with antibiotics. This factor could alter the microbiota. The sample collection was conducted as early as possible in the course of infection. It is reasonable to hypothesize that the observed microbiome alterations occur during the early stages of infection. The nasopharyngeal microbiome is a complex system, and the relationship between specific bacteria and COVID-19 is likely influenced by various factors.

While our results highlight clear trends in the nasopharyngeal microbiome associated with COVID-19 severity, these findings should be interpreted with caution. The relatively small sample size for each clinical group represents a key limitation of this study, which may affect the statistical power and generalizability of the results. Additionally, some methodological choices could have influenced the findings. We used the Greengenes v13.8 database for taxonomic assignment, which, although common, is less up-to-date than databases such as SILVA, potentially limiting classification sensitivity. The closed-reference OTU picking approach, required due to multiplexed sequencing of multiple 16S variable regions, inherently restricts taxonomic resolution and excludes sequences that were absent from the reference database, potentially missing relevant diversity, and preventing resolution at the species or strain level. Rarefaction to equalize sequencing depth led to the exclusion of four samples and may reduce sensitivity to low-abundance taxa, affecting diversity estimates. Importantly, these methods were applied consistently across all groups, supporting valid comparative analyses, though absolute diversity values should be viewed with caution. Nevertheless, despite these constraints, our findings offer valuable insights and establish a foundation for future, larger-scale studies.

Further research is necessary to fully understand the role of the nasopharyngeal microbiome in the onset and severity of COVID-19 to identify potential targets for therapies or preventive measures.

## Data Availability

The datasets presented in this study can be found in online repositories. The names of the repository/repositories and accession number(s) can be found at: https://www.ncbi.nlm.nih.gov/, BioProject: PRJNA1176675. The scripts used for the bioinformatics analyses are available on GitHub at https://github.com/dianalabgr/covid-19_nasopharyngeal_microbiome.
