# Supplementary material for: Identification of nasopharyngeal microbial dysbiosis in COVID-19 patients by 16S rRNA gene sequencing
Source: Front Microbiol. 2025 Aug 29;16:1631198. doi: 10.3389/fmicb.2025.1631198 (PMC12426191; doi:10.3389/fmicb.2025.1631198)
Supplement: Supplementary file 1 [file Supplementary_file_1.docx]

# SUPPLEMENTARY MATERIAL

**Supplementary Table S1:** Taxa median relative abundance per patient group, and differential abundance characterization for each comparison. Differential abundance analysis was performed with the ALDEX2 algorithm and default parameters, as described in the Methods section. For each group comparison, only taxa showing significant differential abundance (expected Benjamini–Hochberg corrected p-value of Welch’s t-test (we.eBH) ≤0.05) are reported. The direction of change (UP or DOWN) indicates an increase or decrease in the group listed first compared to the group listed second. For significant cases (corrected p≤0.05), the corrected p-value is shown after the UP/DOWN label (e.g., UP, p=0.012); for non-significant comparisons, no designation is shown.  Changes also identified as significant in ANCOM analyses are bolded. Only differentially abundant taxa with at least 4% median relative abundance in at least one study group are included in the table. Median relative abundances for each group are displayed in the final columns.

|  | **Differentially abundant taxa** | | | | | | **Median relative % abundance** | | | |
| --- | --- | --- | --- | --- | --- | --- | --- | --- | --- | --- |
| **Taxon** | **in SE compared to NE** | **in MI compared to NE** | **in AS compared to NE** | **in SE compared to AS** | **in MI compared to AS** | **in SE compared to MI** | **NE** | **AS** | **MI** | **SE** |
| ***Phylum level*** | | | | | | | | | | |
| *Fusobacteria* | **DOWN, p=0.000** | **DOWN, p=0.003** | **DOWN, p=0.021** |  |  |  | 7.46 | 0.04 | 0.01 | 0 |
| *Proteobacteria* | UP, p=0.002 | UP, p=0.004 |  |  |  |  | 18.92 | 12.36 | 53.81 | 45.58 |
| *Actinobacteria* |  | **UP, p=0.000** | UP, p=0.015 |  |  | DOWN, p=0.012 | 3.38 | 23.51 | 16.57 | 4.01 |
| *Bacteroidetes* |  | **DOWN, p=0.005** |  | **UP, p=0.039** |  | **UP, p=0.001** | 30.43 | 1.16 | 0.35 | 13.73 |
| *Firmicutes* |  |  | UP, p=0.039 |  |  |  | 29.07 | 38.94 | 17.37 | 33.31 |
| ***Family level*** | | | | | | | | | | |
| *Fusobacteriaceae* | **DOWN, p=0.000** | **DOWN, p=0.001** | **DOWN, p=0.007** |  |  |  | 6.15 | 0.01 | 0.01 | 0 |
| *Pasteurellaceae* | **DOWN, p=0.000** | **DOWN, p=0.000** | **DOWN, p=0.034** |  |  |  | 7.42 | 0.05 | 0.01 | 0 |
| *Prevotellaceae* | **DOWN, p=0.000** | **DOWN, p=0.001** | **DOWN, p=0.009** |  |  |  | 17.83 | 0.09 | 0.13 | 0 |
| *Veillonellaceae* | **DOWN, p=0.003** | **DOWN, p=0.001** | **DOWN, p=0.041** |  |  |  | 8.3 | 0.23 | 0.3 | 0.29 |
| *Streptococcaceae* | **DOWN, p=0.036** | **DOWN, p=0.000** |  |  | DOWN, p=0.031 |  | 9.17 | 5.65 | 0.48 | 0.85 |
| *[Weeksellaceae]* | **UP, p=0.000** |  |  | **UP, p=0.015** |  | **UP, p=0.000** | 0.05 | 0.01 | 0.02 | 11.03 |
| *Bacillaceae* | **UP, p=0.000** |  |  | **UP, p=0.023** |  | **UP, p=0.008** | 0 | 0 | 0.01 | 6.26 |
| *Enterobacteriaceae* | **UP, p=0.000** | **UP, p=0.000** | **UP, p=0.003** |  |  |  | 0 | 0.85 | 12.9 | 25.46 |
| *Enterococcaceae* | **UP, p=0.000** |  |  | **UP, p=0.019** |  | **UP, p=0.000** | 0 | 0 | 0 | 4.47 |
| *Corynebacteriaceae* | UP, p=0.007 | **UP, p=0.000** | UP, p=0.013 |  |  | DOWN, p=0.023 | 0.03 | 13.2 | 12.87 | 1.22 |
| *Pseudomonadaceae* | UP, p=0.045 | **UP, p=0.000** |  |  |  |  | 0 | 0.24 | 4.95 | 0.17 |
| *Alcaligenaceae* |  | **UP, p=0.000** |  |  | **UP, p=0.003** | **DOWN, p=0.001** | 0 | 0 | 14.12 | 0.01 |
| *Halomonadaceae* |  | **UP, p=0.000** |  |  | **UP, p=0.000** | **DOWN, p=0.000** | 0 | 0 | 19.76 | 0 |
| *Staphylococcaceae* |  | **UP, p=0.000** | **UP, p=0.001** |  |  |  | 0.07 | 6.77 | 4.15 | 0.75 |
| ***Genus level*** | | | | | | | | | | |
| *Fusobacterium* | **DOWN, p=0.000** | **DOWN, p=0.001** | **DOWN, p=0.015** |  |  |  | 6.15 | 0.01 | 0.01 | 0 |
| *Haemophilus* | **DOWN, p=0.000** | **DOWN, p=0.000** |  |  |  |  | 6.09 | 0.05 | 0 | 0 |
| *Prevotella* | **DOWN, p=0.000** | **DOWN, p=0.001** | **DOWN, p=0.018** |  |  |  | 17.83 | 0.09 | 0.13 | 0 |
| *Veillonella* | **DOWN, p=0.000** | **DOWN, p=0.000** |  |  |  |  | 7.71 | 0.06 | 0.01 | 0 |
| *Streptococcus* | **DOWN, p=0.002** | **DOWN, p=0.000** |  |  |  |  | 7.35 | 0.59 | 0.27 | 0.01 |
| *Anoxybacillus* | **UP, p=0.000** |  |  | UP, p=0.027 |  | **UP, p=0.000** | 0 | 0 | 0 | 4.93 |
| *Elizabethkingia* | **UP, p=0.000** |  |  | **UP, p=0.025** |  | **UP, p=0.000** | 0 | 0 | 0 | 7.89 |
| *Enterococcus* | **UP, p=0.000** |  |  | UP, p=0.029 |  | **UP, p=0.000** | 0 | 0 | 0 | 4.47 |
| *Serratia* | **UP, p=0.000** | **UP, p=0.000** |  |  |  |  | 0 | 0.16 | 1.9 | 13.08 |
| *Corynebacterium* | UP, p=0.020 | **UP, p=0.000** | UP, p=0.023 |  |  | DOWN, p=0.015 | 0.03 | 13.2 | 12.87 | 1.22 |
| *Citrobacter* |  | **UP, p=0.000** |  |  | **UP, p=0.004** | **DOWN, p=0.000** | 0 | 0 | 4.49 | 0 |
| *Halomonas* |  | **UP, p=0.000** |  |  | **UP, p=0.000** | DOWN, p=0.000 | 0 | 0 | 19.76 | 0 |
| *Pseudomonas* |  | **UP, p=0.000** |  |  | UP, p=0.029 | DOWN, p=0.036 | 0 | 0.18 | 4.92 | 0.17 |
| *Staphylococcus* |  | **UP, p=0.000** | UP, p=0.002 |  |  |  | 0.07 | 6.75 | 4.09 | 0.75 |

**Supplementary Table S2:** Number of reads sequenced (Sequence count) and number of reads attributed to bacteria (Feature count) per sample, sorted by Sequence count in descending order. (*)The four samples with the lowest counts were excluded from further bioinformatics analysis.

| **sample-id** | **Sequence Counts** | **Feature Counts** |  | **sample-id** | **Sequence Counts** | **Feature Counts** |
| --- | --- | --- | --- | --- | --- | --- |
| MI-04 | 238979 | 194309 |  | NE-06 | 50298 | 35452 |
| MI-06 | 223979 | 184828 |  | SE-09 | 50071 | 33038 |
| MI-10 | 189991 | 151173 |  | NE-05 | 48940 | 32650 |
| MI-02 | 180895 | 147116 |  | SE-13 | 48577 | 35730 |
| SE-19 | 180192 | 145112 |  | AS-17 | 48036 | 38274 |
| MI-12 | 132157 | 114024 |  | SE-12 | 47329 | 38146 |
| MI-13 | 131343 | 114738 |  | SE-03 | 45380 | 30598 |
| NE-25 | 130547 | 109685 |  | AS-14 | 44756 | 34989 |
| MI-05 | 126882 | 106963 |  | AS-11 | 44270 | 34821 |
| MI-08 | 124252 | 101997 |  | AS-12 | 43283 | 33841 |
| NE-19 | 121355 | 99440 |  | NE-15 | 42734 | 31477 |
| NE-24 | 118126 | 92948 |  | AS-06 | 42281 | 31608 |
| MI-09 | 117757 | 94680 |  | AS-16 | 41379 | 29657 |
| MI-15 | 117259 | 98421 |  | SE-10 | 40182 | 25654 |
| NE-23 | 114257 | 89740 |  | NE-04 | 39759 | 26220 |
| NE-18 | 112100 | 86492 |  | AS-18 | 39756 | 30521 |
| NE-20 | 111201 | 89367 |  | AS-19 | 38723 | 30627 |
| MI-01 | 111039 | 84672 |  | NE-07 | 38680 | 29748 |
| MI-16 | 110885 | 91850 |  | NE-17 | 38355 | 30758 |
| NE-21 | 107670 | 86226 |  | NE-02 | 38274 | 26067 |
| SE-07 | 96813 | 70662 |  | AS-09 | 38170 | 30847 |
| MI-11 | 93052 | 76286 |  | NE-11 | 37945 | 28668 |
| MI-03 | 91451 | 73857 |  | SE-11 | 37686 | 24496 |
| SE-01 | 87203 | 71562 |  | AS-05 | 37609 | 25977 |
| NE-26 | 81101 | 66549 |  | NE-16 | 37313 | 29303 |
| MI-07 | 79994 | 63013 |  | NE-10 | 36228 | 27935 |
| SE-06 | 73212 | 51565 |  | SE-18 | 36223 | 28002 |
| NE-01 | 64578 | 48672 |  | AS-04 | 35736 | 24582 |
| SE-17 | 63414 | 50520 |  | AS-13 | 35634 | 26699 |
| NE-22 | 62781 | 50159 |  | AS-03 | 35292 | 25542 |
| NE-03 | 60187 | 42948 |  | NE-13 | 34347 | 26636 |
| AS-01 | 59420 | 43749 |  | AS-15 | 32860 | 26338 |
| SE-16 | 59383 | 50607 |  | AS-08 | 32245 | 21432 |
| SE-05 | 59380 | 40705 |  | AS-02 | 31838 | 23101 |
| AS-07 | 59202 | 46018 |  | SE-14 | 31268 | 23408 |
| NE-14 | 58962 | 47040 |  | NE-12 | 29527 | 22451 |
| NE-08 | 58107 | 45429 |  | SE-04* | 24705 | 15639 |
| AS-10 | 56938 | 43758 |  | SE-15* | 23505 | 18367 |
| MI-14 | 54760 | 44501 |  | NE-27* | 22048 | 17201 |
| NE-09 | 54082 | 41821 |  | SE-08* | 17061 | 11089 |
| SE-02 | 52785 | 36373 |  |  |  |  |


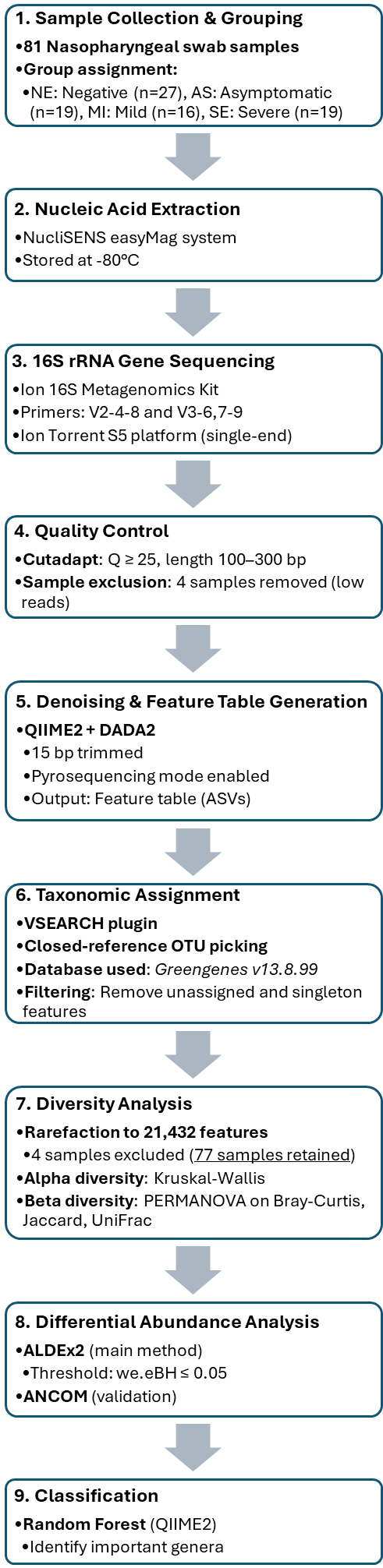


**Supplementary Figure S1**: Analytical workflow for nasopharyngeal microbiome analysis. Overview of the experimental and computational pipeline used in this study. The diagram outlines key steps including sample collection and clinical grouping, nucleic acid extraction, 16S rRNA gene sequencing, quality control, denoising and feature table generation (QIIME2 + DADA2), taxonomic assignment (VSEARCH with Greengenes database), diversity analysis, differential abundance testing (ALDEx2 and ANCOM), and classification using a random forest model.


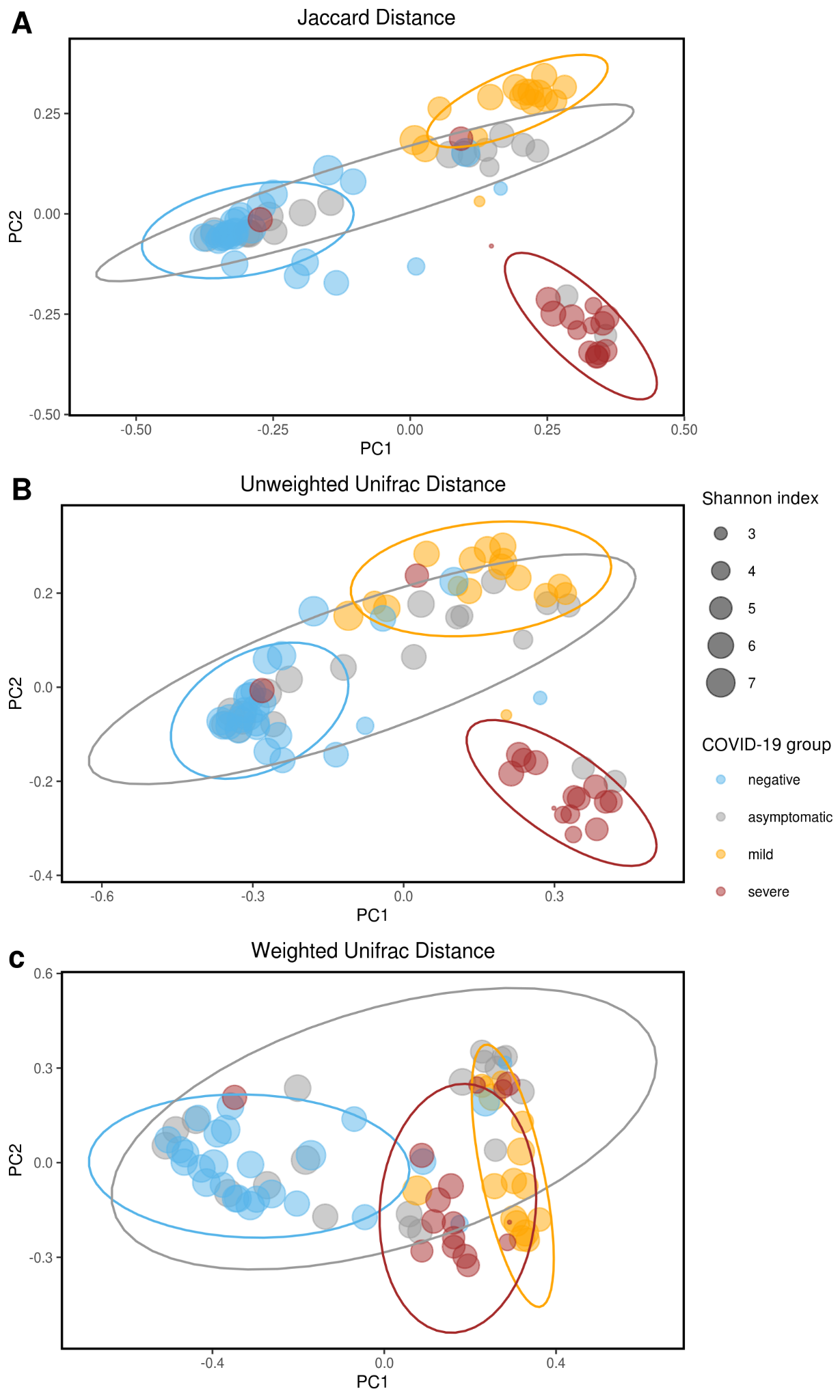


**Supplementary Figure S2:** Composition dissimilarity between the 4 patient groups as indicated by the Jaccard (a), Unweighted UniFrac (b) and Weighted Unifrac (c) distances, depicted with two Principal Components (PERMANOVA p<0.01, q<0.01 for all pairwise comparisons).

**Supplementary Table S3:** All four beta-diversity measures were significantly different between the four groups (Q-values<0.01, PERMANOVA)

| **Bray Curtis, Pairwise PERMANOVA results** | | | | | | |
| --- | --- | --- | --- | --- | --- | --- |
| **Group 1** | **Group 2** | **Sample size** | **Permutations** | **pseudo-F** | **p-value** | **q-value** |
| asymptomatic | mild | 35 | 999 | 6.617715 | 0.001 | 0.0012 |
|  | negative | 45 | 999 | 2.800187 | 0.002 | 0.002 |
|  | severe | 35 | 999 | 4.582393 | 0.001 | 0.0012 |
| mild | negative | 42 | 999 | 13.86062 | 0.001 | 0.0012 |
|  | severe | 32 | 999 | 10.05841 | 0.001 | 0.0012 |
| negative | severe | 42 | 999 | 9.508864 | 0.001 | 0.0012 |
| **Jaccard distance, Pairwise PERMANOVA results** | | | | | | |
|  |  | **Sample size** | **Permutations** | **pseudo-F** | **p-value** | **q-value** |
| **Group 1** | **Group 2** |  |  |  |  |  |
| asymptomatic | mild | 35 | 999 | 3.677931 | 0.001 | 0.001 |
|  | negative | 45 | 999 | 2.575316 | 0.001 | 0.001 |
|  | severe | 35 | 999 | 4.403632 | 0.001 | 0.001 |
| mild | negative | 42 | 999 | 8.414675 | 0.001 | 0.001 |
|  | severe | 32 | 999 | 6.536905 | 0.001 | 0.001 |
| negative | severe | 42 | 999 | 9.166323 | 0.001 | 0.001 |
| **Unweighted Unifrac, Pairwise PERMANOVA results** | | | | | | |
| **Group 1** | **Group 2** | **Sample size** | **Permutations** | **pseudo-F** | **p-value** | **q-value** |
| asymptomatic | mild | 35 | 999 | 3.809729 | 0.001 | 0.001 |
|  | negative | 45 | 999 | 3.999245 | 0.001 | 0.001 |
|  | severe | 35 | 999 | 6.422655 | 0.001 | 0.001 |
| mild | negative | 42 | 999 | 11.86056 | 0.001 | 0.001 |
|  | severe | 32 | 999 | 7.404302 | 0.001 | 0.001 |
| negative | severe | 42 | 999 | 16.68989 | 0.001 | 0.001 |
| **Weighted Unifrac, Pairwise PERMANOVA results** | | | | | | |
| **Group 1** | **Group 2** | **Sample size** | **Permutations** | **pseudo-F** | **p-value** | **q-value** |
| asymptomatic | mild | 35 | 999 | 7.396076 | 0.002 | 0.0024 |
|  | negative | 45 | 999 | 4.824469 | 0.004 | 0.004 |
|  | severe | 35 | 999 | 4.805435 | 0.002 | 0.0024 |
| mild | negative | 42 | 999 | 26.31655 | 0.001 | 0.002 |
|  | severe | 32 | 999 | 6.000054 | 0.001 | 0.002 |
| negative | severe | 42 | 999 | 15.47945 | 0.001 | 0.002 |


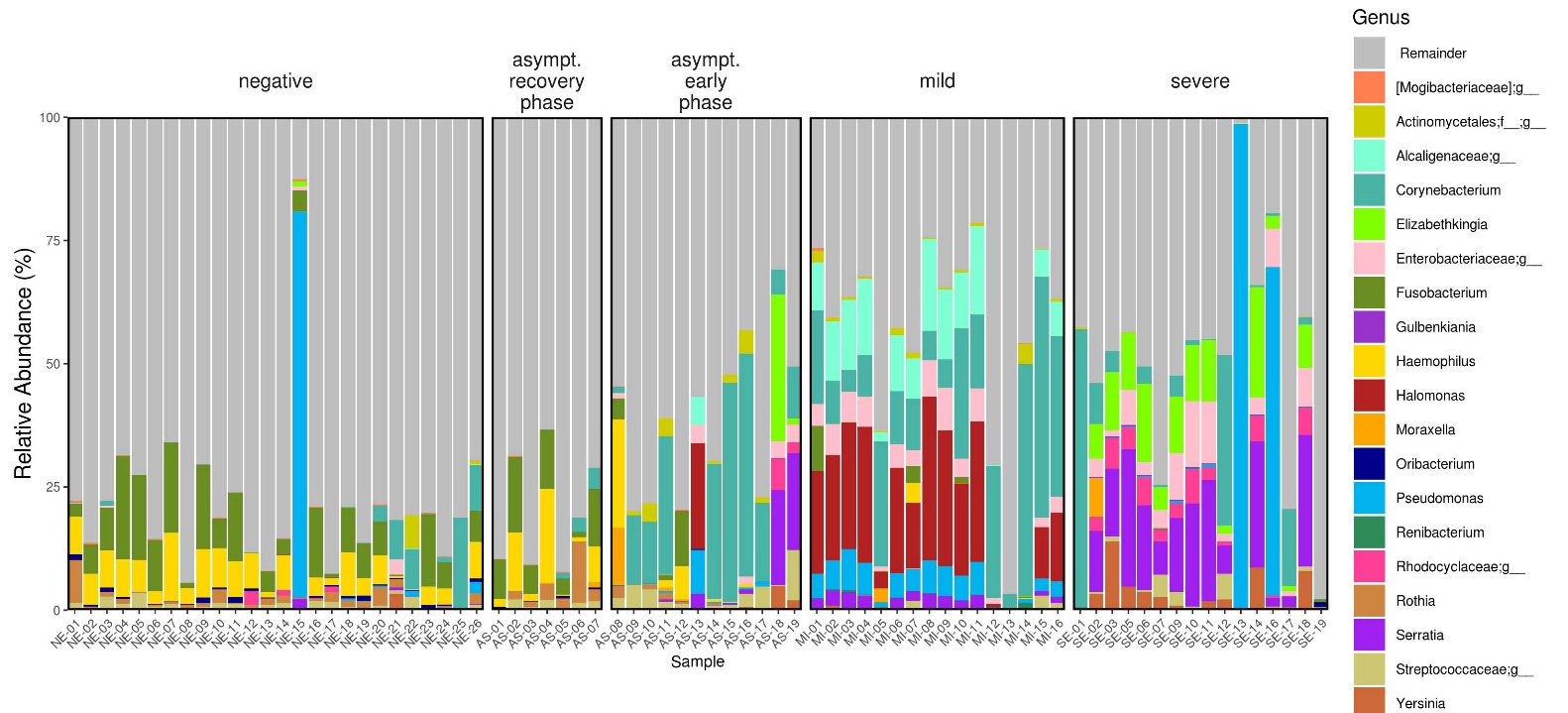


**Supplementary Figure S3:** Relative abundance bar plot of the top 19 genera identified as most significant by the random forest classifier model (as listed in Table 1).


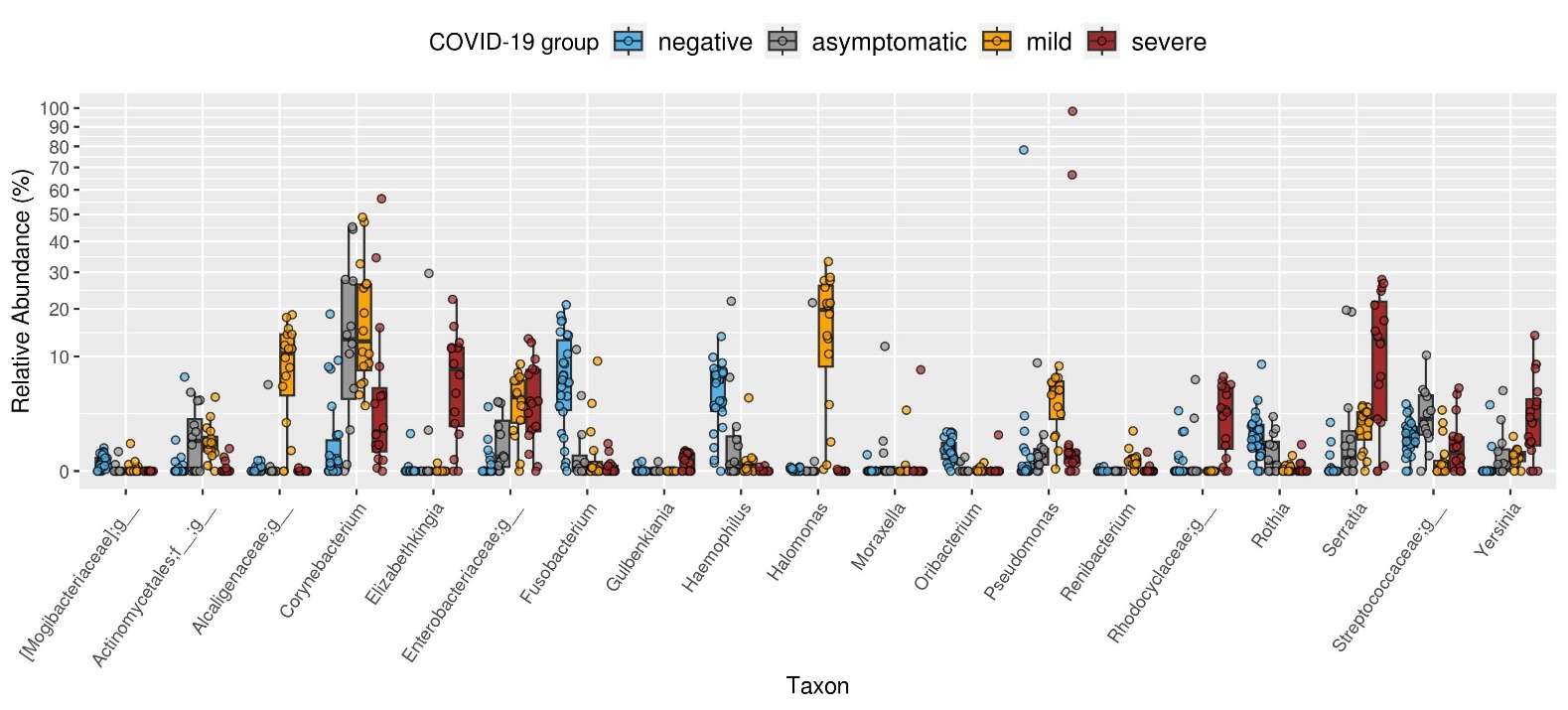


**Supplementary Figure S4:** Relative abundance box plot of the top 19 genera identified as most significant by the random forest classifier model (as listed in Table 1).
